# Microbiome‐Endocannabinoidome Crosstalk as an Immunometabolic Rheostat in Inflammatory Disease

**DOI:** 10.1096/fba.2026-00201

**Published:** 2026-10-15

**Authors:** Reuben S. Maghembe, Donath Damian, Samweli Bahati, Abdalah Makaranga

**Affiliations:** ^1^ Department of Microbiology and Parasitology, Faculty of Medicine St. Francis University College of Health and Allied Sciences Ifakara Tanzania; ^2^ Department of Omics and Computational Biology AfroBiomics Co. Ltd. Dar es Salaam Tanzania; ^3^ Department of Biochemistry, Mbeya College of Health and Allied Sciences University of Dar es Salaam Mbeya Tanzania; ^4^ Department of Agriculture, Earth and Environmental Sciences (DAES), Faculty of Science Mwenge Catholic University (MWECAU) Moshi Tanzania

**Keywords:** endocannabinoidome, engineered probiotics, epithelial barrier, immunometabolic rheostat, inflammation, microbiome, N‐acylethanolamines, precision medicine

## Abstract

Chronic inflammatory diseases reflect disrupted coordination among microbial ecology, epithelial barriers, lipid signaling, and immunity. The microbiome‐endocannabinoidome (eCBome) axis is already recognized as a regulator of metabolism, permeability, and inflammatory tone; the unresolved issue is how to distinguish direct crosstalk from parallel or associative changes. This review therefore operationalizes the axis as an immunometabolic rheostat defined by five measurable domains: microbial functions and metabolites, compartment‐specific eCBome ligands and enzymes, barrier integrity and microbial translocation, immune‐cell programming, and tissue or clinical outcomes. We distinguish classical endocannabinoids (anandamide and 2‐arachidonoylglycerol) from the endocannabinoid‐related N‐acylethanolamines palmitoylethanolamide and oleoylethanolamide, which act prominently through PPARα, TRPV1, GPR119, and related pathways. Evidence is appraised as direct causal, mechanistically supportive, associative, or hypothesis‐generating. The strongest causal foundation links microbiota manipulation to intestinal eCBome tone, permeability, and metabolic endotoxemia, while disease‐specific evidence is most developed in obesity‐associated inflammation and inflammatory bowel disease. Engineered *Lactobacillus paracasei* platforms that produce PEA or OEA in situ provide particularly informative proof‐of‐concept because they couple a defined microbial perturbation to lipid‐mediator release, receptor‐dependent barrier effects, and inflammatory or metabolic outcomes. By contrast, evidence in MASLD/MASH, neuroinflammation, and systemic autoimmunity remains more compartment‐dependent and heterogeneous. Clinical translation must account for the psychiatric toxicity that ended centrally active CB1 antagonism with rimonabant, the target and off‐target complexity of FAAH/MAGL inhibition, and limitations of surrogate barrier biomarkers. A causal roadmap should combine gnotobiotic and loss‐of‐function models, isotope tracing, organoids, and gut‐on‐chip systems with tissue‐resolved lipidomics and longitudinal, biomarker‐enriched human trials.

## Graphical abstract

The microbiome‐eCBome axis is operationalized across five measurable domains: microbial functions, compartment‐specific lipid signaling, barrier/translocation, immune‐cell programming, and tissue outcomes. Engineered PEA‐ and OEA‐producing bacteria provide causal proof‐of‐concept, while translation requires tissue‐resolved lipidomics, validated barrier assays, receptor/enzyme‐specific models, and safety‐aware human studies.

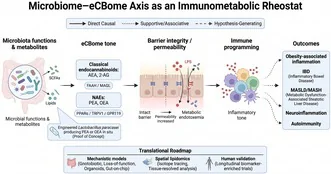

## 1 Introduction

Chronic inflammatory disease emerges when protective immune responses fail to resolve and become coupled to persistent epithelial, metabolic, and tissue stress [1]. Across inflammatory bowel disease (IBD), obesity‐associated inflammation, metabolic dysfunction‐associated steatotic liver disease and steatohepatitis (MASLD/MASH), and selected neuroimmune or autoimmune conditions, microbial imbalance and altered lipid signaling can reinforce barrier dysfunction and immune activation. The microbiome‐eCBome axis is therefore biologically important, but its existence is not itself novel. The central question is how to define when this axis acts as a causal regulator rather than a descriptive association.

The microbiome contributes metabolites, transformed dietary substrates, and immune‐educational signals that influence local and systemic physiology [2, 3, 4, 5, 6]. Taxa associated with SCFA production, mucus interactions, or probiotic activity can support barrier and immune homeostasis [7, 8, 9, 10, 11], whereas dysbiotic states may reduce beneficial functions and increase translocation‐prone or inflammatory outputs [3, 4, 5, 6]. These labels are context dependent: strain traits, diet, geography, medications, host immunity, and disease stage determine whether a taxon or function is beneficial, neutral, or harmful [12, 13, 14, 15]. Accordingly, this review prioritizes microbial functions and measured metabolites over universal taxonomic classifications.

The eCBome extends the classical endocannabinoid system (ECS) beyond CB1, CB2, anandamide (AEA), 2‐arachidonoylglycerol (2‐AG), FAAH, and MAGL to a wider network of lipid mediators and targets [3, 16, 17]. This terminology requires precision. AEA and 2‐AG are classical endocannabinoids. Palmitoylethanolamide (PEA) and oleoylethanolamide (OEA) are endocannabinoid‐related N‐acylethanolamines (NAEs), not interchangeable substitutes for AEA or 2‐AG, and their prominent actions include PPARα, TRPV1, GPR119, and indirect modulation of cannabinoid signaling (Figure 1).

**FIGURE 1:**
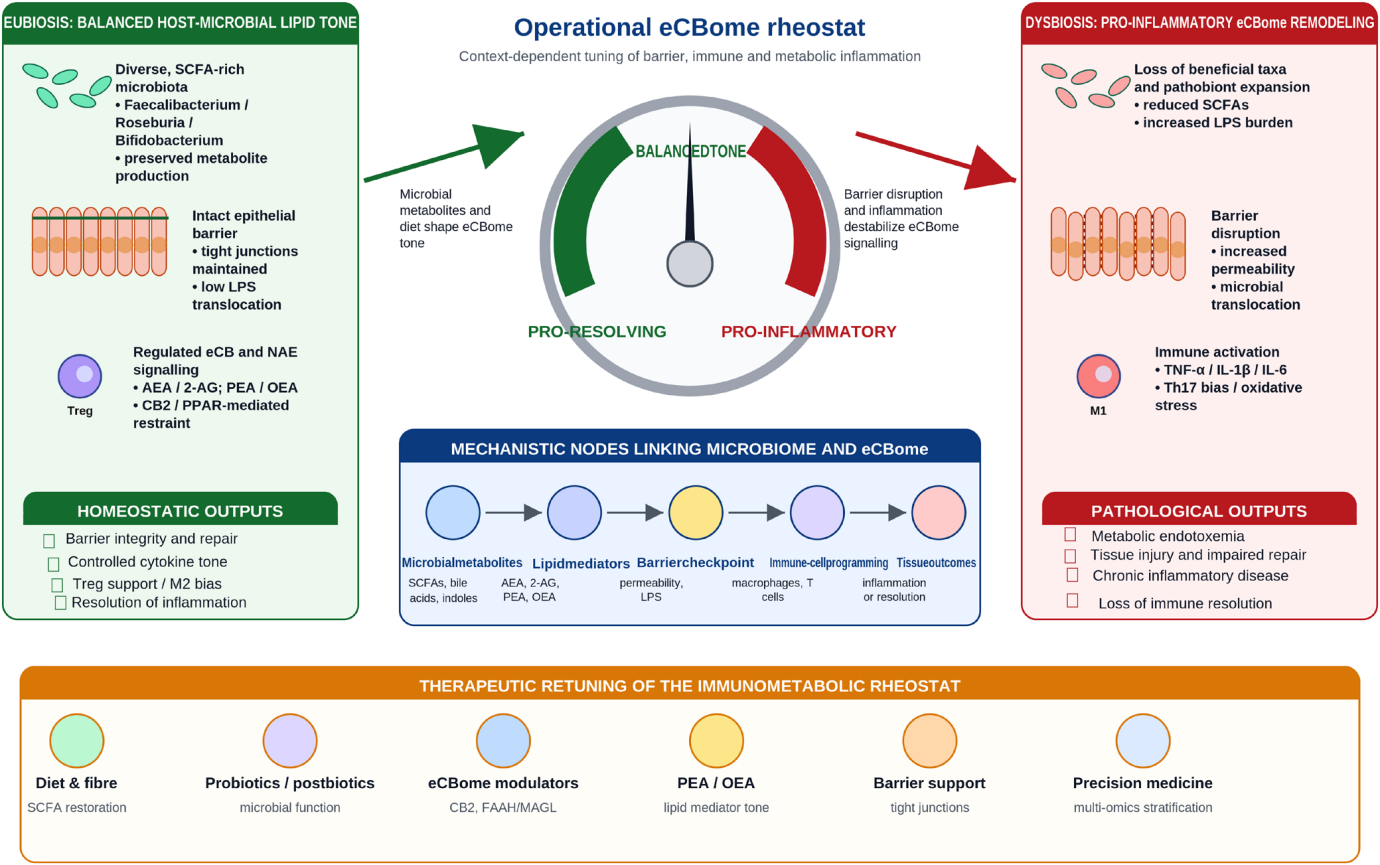
Operational model of the microbiome‐endocannabinoidome immunometabolic rheostat. The rheostat is defined by five measurable domains: Microbial functions and metabolites; compartment‐specific eCBome ligands, enzymes, and receptors; epithelial permeability and microbial translocation; immune‐cell programming; and tissue or clinical outcomes. Direction, magnitude, anatomical compartment, timing, and reversibility determine whether the net state is pro‐resolving, balanced, or pro‐inflammatory.

### 1.1 Review Approach and Evidence Appraisal

This structured narrative review was updated through 24 July 2026 using PubMed/MEDLINE and Google Scholar, supplemented by backward citation tracing from primary studies and field‐defining reviews. Search combinations included microbiome or gut microbiota with endocannabinoidome, endocannabinoid system, AEA, 2‐AG, PEA, OEA, FAAH, MAGL, intestinal permeability, endotoxemia, IBD, obesity, MASLD/MASH, neuroinflammation, engineered probiotic, live biotherapeutic, and lipidomics. Priority was given to primary mechanistic studies, human tissue or intervention data, receptor‐ or enzyme‐dependent models and studies measuring both microbial and eCBome variables. This was not a systematic review or meta‐analysis; evidence was graded by directness, human relevance, intervention strength, compartmental resolution and reproducibility (Table 1).

**TABLE 1:** Core endocannabinoidome components and compartment‐dependent interpretation.

Component	Classification/principal targets	Interpretive relevance
CB1	Classical cannabinoid receptor	Central: appetite, reward and neuropsychiatric effects; peripheral: gut motility, permeability, liver, and adipose metabolism
CB2	Classical cannabinoid receptor	Immune‐cell trafficking, cytokine regulation, and tissue‐dependent inflammatory responses
AEA	Classical endocannabinoid; CB1/CB2, TRPV1	Short‐lived ligand whose meaning depends on tissue production, FAAH activity, and receptor distribution
2‐AG	Classical endocannabinoid; CB1/CB2	Abundant signaling lipid regulated by DAGL and MAGL; also linked to downstream arachidonic‐acid pathways
FAAH	Hydrolase for AEA and several NAEs	Inhibition alters multiple fatty‐acid amides; selectivity and off‐target lipid effects require measurement
MAGL	Principal 2‐AG hydrolase	Controls 2‐AG and arachidonic‐acid availability; effects may differ by tissue and inflammatory state
PEA	Endocannabinoid‐related NAE; PPARα, indirect/other targets	Inflammatory restraint, mast‐cell and macrophage regulation, oxidative stress, and barrier repair
OEA	Endocannabinoid‐related NAE; PPARα, TRPV1, GPR119	Satiety, metabolic regulation, epithelial integrity, and microbiota‐gut‐brain communication
PPARα/γ	Nuclear lipid‐sensing receptors	Metabolic, epithelial and immune transcriptional programs; not cannabinoid receptors
TRPV1	Ion channel	Pain, epithelial signaling, and context‐dependent effects of AEA/OEA/PEA‐related pathways
GPR55/GPR119	Lipid‐sensitive GPCRs	Gut endocrine, metabolic, and immune signaling; ligand specificity remains context dependent

## 2 Operationalizing the Endocannabinoidome Rheostat

The classical ECS comprises CB1 and CB2 receptors, AEA and 2‐AG, and their principal biosynthetic and degradative enzymes, including NAPE‐PLD, DAGL, FAAH, and MAGL [16, 17, 18]. The eCBome broadens this architecture to related NAEs, monoacylglycerols, oxylipins, and lipid‐sensitive targets [3, 16, 17]. PEA is best interpreted as a PPARα‐linked and tissue‐protective NAE with effects on mast cells, macrophages, oxidative stress, and barrier repair. OEA has distinct roles in satiety, metabolic signaling, intestinal permeability, and gut‐brain communication through PPARα, TRPV1, GPR119, and related mechanisms. Their effects can overlap, but their substrates, tissue distributions, and dominant targets differ. The same ligand may also generate different outcomes according to receptor abundance, enzyme activity, and cell type [19, 20, 21].

The rheostat concept adds value only when it is operational rather than metaphorical. We define it as a multidimensional state that can be measured across five domains: (i) microbial genes, taxa, and metabolites; (ii) tissue‐specific eCBome ligands, receptors, and enzymes; (iii) barrier integrity and microbial translocation; (iv) immune‐cell phenotype and immunometabolism; and (v) tissue injury, resolution, and clinical outcome [3, 19, 22]. Direct crosstalk requires a microbial perturbation that changes a defined eCBome component and a downstream phenotype. Mechanistically supportive convergence indicates that microbial and lipid pathways affect the same outcome without proving a causal chain. Associative evidence identifies co‐variation, whereas hypothesis‐generating evidence extrapolates across tissues or diseases (Box 1).

BOX 1 **Evidence categories used in this review.**

Direct causal evidence: a controlled microbial perturbation changes a measured eCBome component and a linked phenotype, with receptor, enzyme, or bacterial‐function dependence where feasible. Mechanistically supportive evidence: microbial and eCBome pathways converge on the same outcome, but mediation is incomplete. Associative evidence: microbial and lipid variables co‐vary without established directionality. Hypothesis‐generating evidence: a mechanism is extrapolated across compartments, models, or diseases.

## 3 Microbial Regulation of Host Lipid Signaling: Direct and Indirect Routes

Microbial regulation of the eCBome can occur through several routes, but these routes are not equally established. Direct evidence requires controlled microbiota manipulation, measurement of a host eCBome component and demonstration of a linked physiological effect [2, 3, 23]. Indirect evidence includes microbial metabolites that reshape epithelial or metabolic conditions in which eCBome signaling operates, even when the intermediate lipid pathway is not measured. This distinction prevents functional convergence from being overstated as direct molecular crosstalk (Table 2).

**TABLE 2:** Routes linking microbial biology to the endocannabinoidome.

Linkage route	Example	Evidence interpretation
Direct microbiota‐eCBome regulation	Microbiota manipulation changes intestinal ECS tone, permeability and plasma LPS	Direct causal anchor when microbial perturbation, lipid signaling and phenotype are measured together
Engineered mediator delivery	NAPE‐PLD‐expressing *L. paracasei* produces PEA or OEA in situ	Direct microbial‐to‐lipid intervention; receptor dependence strengthens causality
Substrate remodeling	Dietary fatty acids and microbially transformed lipids alter precursor availability	Mechanistic support; requires isotope or flux tracing to prove source‐product linkage
Metabolite convergence	SCFAs, bile acids, and indoles regulate barrier and immune pathways	Indirect unless eCBome components are measured and mediate the phenotype
Translocation feedback	LPS exposure activates innate immunity and can alter host lipid metabolism	Bidirectional mechanism; barrier injury may be cause, consequence, or amplifier
Associative co‐variation	Microbiome composition correlates with plasma or fecal NAEs	Hypothesis‐generating unless temporality, compartment, and confounding are resolved

SCFAs generated by fiber fermentation support colonocyte metabolism, tight‐junction integrity, and regulatory immune programs [4, 5, 6, 24]. They are not endocannabinoids and should not be presented as direct eCBome ligands. Their relevance is primarily contextual: SCFAs can alter epithelial energy status, inflammatory signaling, and substrate availability, thereby changing the biological environment in which eCBome pathways act (Figure 2).

**FIGURE 2:**
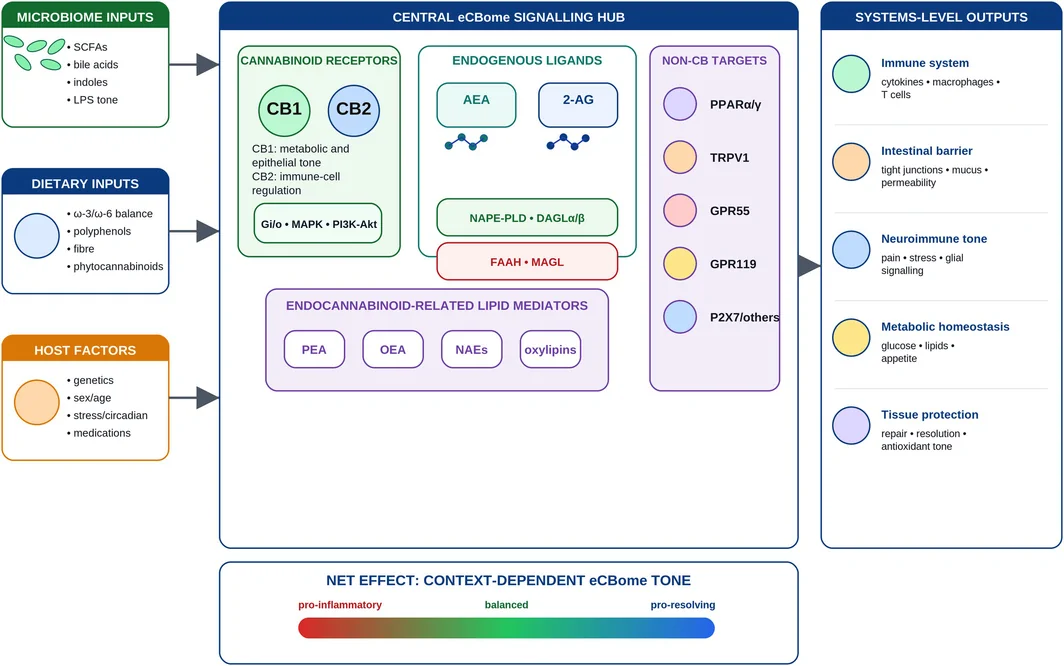
Molecular architecture and terminology of the expanded endocannabinoidome. Classical endocannabinoids comprise AEA and 2‐AG with CB1/CB2 and their metabolic enzymes. PEA and OEA are endocannabinoid‐related NAEs with prominent actions through PPARα, TRPV1, GPR119, and other non‐CB targets. Microbial, dietary, and host inputs shape these components in a tissue‐ and cell‐specific manner.

Microbially transformed bile acids provide a second route. Through FXR, TGR5, and enterohepatic signaling, secondary bile acids influence lipid metabolism, epithelial function, and inflammation [25, 26, 27, 28]. Recent causal work with *Christensenella minuta* illustrates how a defined bacterial function can generate acylated secondary bile acids, alter intestinal FXR signaling, and improve metabolic phenotypes, but this remains mechanistically adjacent to, rather than proof of, direct eCBome modulation.

Microbial tryptophan metabolites and indoles activate aryl hydrocarbon receptor pathways that support epithelial repair and immune tolerance [29, 30, 31, 32]. As with SCFAs, this is best classified as mechanistically supportive convergence unless eCBome ligands, receptors, or enzymes are measured within the same causal experiment.

The strongest direct anchor remains the demonstration that gut microbiota can regulate intestinal ECS tone, permeability and plasma LPS, linking microbial ecology to adipogenesis and metabolic endotoxemia [22, 33, 34]. Dietary lipids simultaneously shape microbial communities, bile‐acid pools and fatty‐acid substrates available for AEA, 2‐AG, and NAE synthesis [35, 36, 37, 38, 39]. Here, “microbial lipid remodeling” denotes microbial transformation of dietary or host lipids, bile acids and fatty‐acid substrates that change host lipid‐mediator production, turnover, or receptor engagement; it should not be used as a generic synonym for dysbiosis.

## 4 Tissue Compartmentalization, Barrier Biology, and Measurement

The biological meaning of an eCBome measurement depends on its compartment. Intestinal mucosa contains epithelial, immune, neural, and stromal sources; fecal lipids reflect luminal content, microbial metabolism, diet, and host secretion; plasma integrates production and clearance from multiple organs; portal blood preferentially samples gut‐derived signals; and liver, adipose tissue, and brain have distinct receptor and enzyme profiles [19, 40, 41, 42, 43]. A circulating NAE concentration therefore cannot be assumed to represent mucosal receptor engagement or central signaling (Figure 3).

**FIGURE 3:**
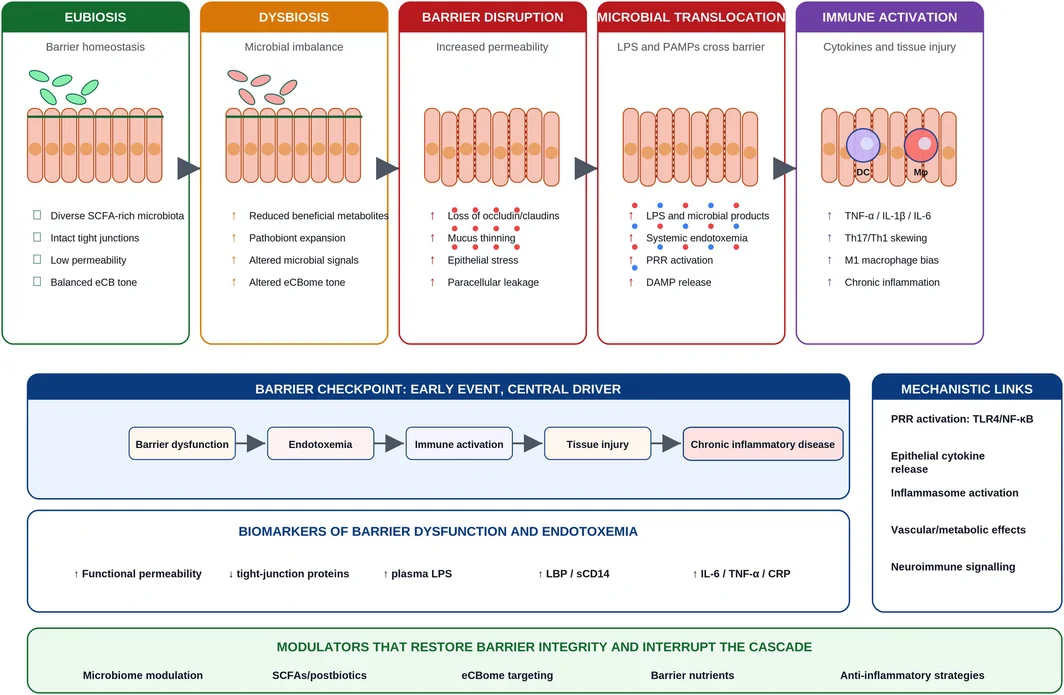
Barrier and translocation checkpoint. Microbial functions and compartment‐specific eCBome signaling converge on mucus, epithelial junctions, and epithelial stress responses. Barrier failure can permit microbial products to engage innate immune pathways, but permeability, enterocyte injury, endotoxin exposure, and monocyte activation require distinct measurements rather than interchangeable surrogate biomarkers.

### 4.1 The Epithelial Barrier as a Testable Checkpoint

Barrier dysfunction offers a plausible route from dysbiosis to systemic inflammation, but the sequence is not universal. Reduced beneficial metabolites, mucus disruption, epithelial energy failure, and inflammatory injury may increase permeability, permitting LPS and other microbial products to reach immune and metabolic tissues [22, 33, 34, 41, 42, 43, 44]. Direct permeability assays and tissue measurements are therefore more informative than inferring a leaky barrier from systemic inflammation alone.

Within the intestine, CB1 and CB2 have different anatomical and functional implications. Peripheral epithelial or enteric CB1 signaling can influence motility, secretion, permeability, and metabolic sensing, whereas central CB1 signaling strongly affects appetite, reward, and neuropsychiatric function. CB2 is enriched in immune compartments and more consistently linked to leukocyte trafficking and cytokine regulation [19, 22]. These distinctions are essential when interpreting receptor‐targeted interventions.

PEA and OEA provide nonclassical routes to barrier regulation. In intestinal models, both can modify permeability through PPARα‐ and TRPV1‐associated mechanisms, while PEA additionally supports inflammatory restraint and tissue protection and OEA links epithelial integrity to metabolic and satiety signaling [19, 20, 21, 22, 23, 45, 46, 47]. Their concentrations, targets, and effects should therefore be reported separately rather than collapsed into a single “eCB‐like” category.

Barrier biomarkers require critical interpretation. Lactulose‐mannitol or related multi‐sugar tests assess functional permeability, whereas intestinal fatty‐acid‐binding protein reflects enterocyte injury. LPS, LPS‐binding protein (LBP), and soluble CD14 (sCD14) index different aspects of microbial exposure or innate immune activation and are not interchangeable measures of permeability. Commercial zonulin ELISAs may detect proteins other than pre‐haptoglobin 2 and can correlate poorly with functional permeability, so zonulin alone should not establish barrier failure [48, 49].

Sex, age, diet, circadian state, medications, and sampling conditions add further variation. Human studies report sex‐ and age‐associated differences in gut microbial diversity and barrier responses, and targeted lipidomics has identified sex‐ and age‐specific eCBome profiles in some disease settings [19, 22, 40, 50, 51]. Future studies should prespecify these variables and use matched compartments, standardized fasting and collection procedures, and rapid sample stabilization [3, 19, 22].

## 5 Immune‐Cell Programming by Microbiome‐eCBome Signals

Microbial products and lipid mediators become disease‐relevant when they alter immune‐cell state. Macrophages, dendritic cells, T cells, and neutrophils integrate pattern‐recognition signals, tissue metabolites, and eCBome ligands through pathways that influence cytokine production, migration, phagocytosis, antigen presentation, and repair [19, 20, 21, 52, 53]. Evidence is strongest when these cellular effects are demonstrated after a defined microbial or lipid perturbation rather than inferred from parallel disease‐associated changes.

Macrophages integrate LPS, bile‐acid signals, NAEs, and tissue‐derived danger cues. CB2 and PPAR pathways can modify inflammatory cytokines, oxidative stress, and repair programs [20, 21, 52, 53, 54, 55, 56, 57]. T‐cell responses are shaped by SCFAs, indoles, and antigen‐presenting‐cell signals; the balance between regulatory T cells and Th17 programs is especially relevant at mucosal surfaces [5, 29, 30, 58, 59, 60, 61, 62]. These interactions illustrate convergence, but direct microbiome‐to‐eCBome‐to‐T‐cell chains remain less frequently tested.

Dendritic cells connect epithelial sampling to adaptive immunity, and microbial metabolites can alter their maturation and tolerogenic capacity [29, 30, 58, 63]. Neutrophils contribute to antimicrobial defense and collateral injury through reactive oxygen species, proteases, and extracellular traps; eCBome‐related lipids may influence recruitment and resolution, although this evidence is less mature.

A causal immune framework should therefore measure both the input and the responding cell. Examples include receptor‐deficient macrophages exposed to defined microbial metabolites, antigen‐presenting‐cell and T‐cell co‐cultures with controlled lipid supplementation, and engineered bacteria coupled to cell‐specific receptor blockade. Such designs distinguish direct receptor dependence from nonspecific changes in inflammation [21, 52, 53, 64].

Immunometabolism provides the integrating layer. Pro‐inflammatory macrophages and activated lymphocytes reprogram glucose, lipid, and mitochondrial metabolism to support effector function, while resolution and repair require different metabolic states [52, 53, 59]. Microbial metabolites and eCBome mediators can therefore influence inflammation both through receptor signaling and through the substrates available to immune cells.

The most defensible interpretation is that the microbiome‐eCBome axis modulates immune‐cell programming in a cell‐, tissue‐, and disease‐stage‐specific manner [19, 21, 52, 53, 58, 59]. Broad statements that the axis is uniformly anti‐inflammatory or pro‐inflammatory obscure this heterogeneity.

## 6 Disease‐Specific Evidence Modules

### 6.1 Inflammatory Bowel Disease

IBD is the most anatomically direct model because microbial communities, mucosal eCBome pathways, epithelial barriers, and immune cells coexist at the site of disease. Crohn disease and ulcerative colitis show altered microbial functions, mucosal inflammation, barrier injury, and changes in tissue or circulating eCBome mediators [5, 19, 40, 59, 65, 66, 67, 68]. Human studies demonstrate association and compartment‐specific dysregulation, whereas causal evidence is still derived mainly from experimental colitis and engineered microbial systems (Figure 4).

**FIGURE 4:**
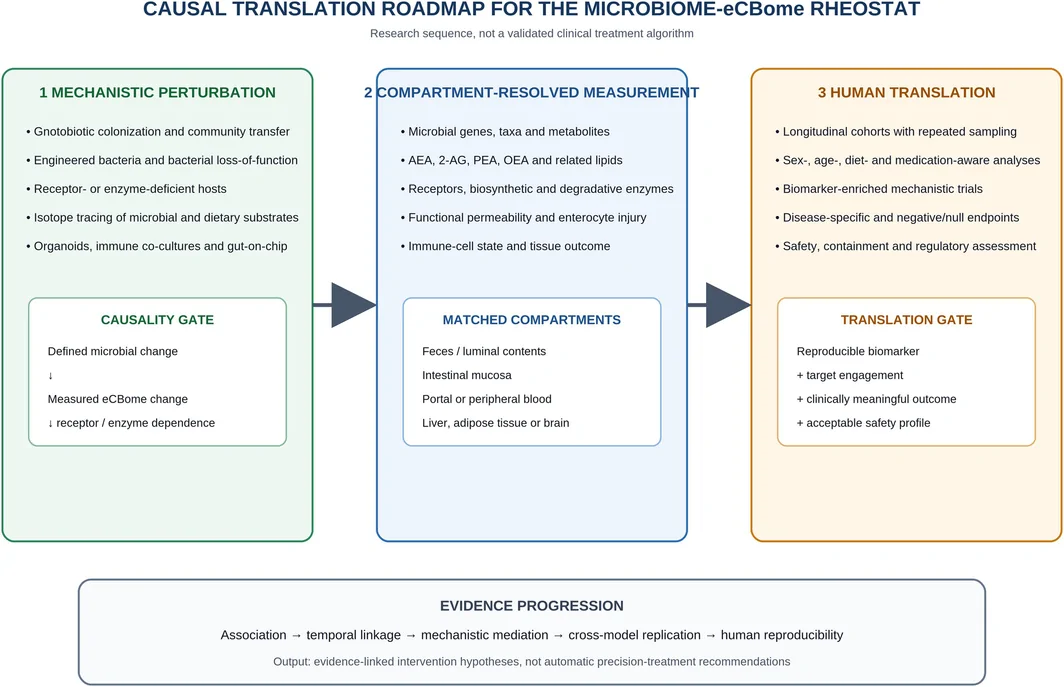
Causal translation roadmap. The figure presents a research sequence rather than a validated treatment algorithm: Mechanistic perturbation in gnotobiotic, engineered‐microbial, and receptor‐deficient systems; compartment‐resolved metagenomic, lipidomic, barrier, and immune measurements; replication across sex, diet, and disease stage; and biomarker‐enriched longitudinal human trials with safety and regulatory gates.

Therapeutic interpretation must separate symptom relief from disease modification. Cannabinoid exposure may improve pain, appetite, or quality of life, but controlled studies have not consistently demonstrated mucosal healing or durable suppression of intestinal inflammation [19, 59, 65, 66, 69, 70, 71]. PEA‐producing engineered *L. paracasei* provides a more mechanistically resolved model: bacterial NAPE‐PLD expression generated PEA in situ, improved tight‐junction proteins and inflammatory injury, and lost protection in PPARα‐deficient mice [72]. This is direct proof‐of‐concept in animals, not yet clinical efficacy [59, 66, 69, 70, 73].

### 6.2 Obesity and Metabolic Inflammation

Obesity‐associated inflammation has the strongest systems‐level support. High‐fat feeding can alter microbiota, permeability, and LPS exposure, while adipose macrophage activation and insulin resistance provide measurable systemic outcomes [22, 33, 34, 36, 69, 74, 75]. The foundational microbiota‐ECS study directly connected intestinal endocannabinoid tone with permeability, plasma LPS, and adipogenesis, although translation to heterogeneous human obesity requires caution.

CB1 signaling contributes centrally to appetite and reward and peripherally to intestinal, hepatic, and adipose metabolism [22, 36, 69, 74]. Other eCBome components may have opposing or compensatory effects [36, 69]. An engineered OEA‐producing *L. paracasei* platform improved duodenal barrier function, microbiota composition, metabolic abnormalities, and neurobehavioral phenotypes in high‐fat‐diet‐fed male mice, with little benefit from the native strain alone [76]. The study strengthens causal plausibility but is limited to one sex, one model, and a regulated oleate‐supplemented bacterial system.

### 6.3 MASLD/MASH and the Gut‐Liver Axis

MASLD/MASH extends the framework from intestinal exposure to portal and hepatic compartments. Increased permeability and altered microbial metabolites can reach the liver, where hepatocytes, Kupffer cells, stellate cells, and endothelial cells contribute to steatosis, inflammation, and fibrosis [28, 77, 78, 79, 80, 81, 82]. The nomenclature MASLD/MASH is used here; older NAFLD/NASH terminology is retained only when referring to historical studies or titles.

Hepatic CB1 signaling is linked to lipogenesis and steatosis, whereas CB2 effects vary with cell type and disease stage [36, 77, 78]. Plasma eCBome measurements cannot substitute for portal or hepatic tissue profiling, and fecal microbial associations do not establish a direct microbial‐eCBome mechanism in the liver.

Accordingly, evidence for a gut‐liver microbiome‐eCBome pathway is moderate: each component is biologically credible, but few human studies measure microbial function, portal or hepatic lipid mediators, receptor activity, and longitudinal fibrosis outcomes together [28, 77, 78].

### 6.4 Systemic Extensions: Neuroinflammation and Autoimmunity

The gut‐brain axis can transmit microbial and inflammatory signals through metabolites, vagal pathways, endocrine responses, and circulating immune mediators, while the eCBome regulates stress, pain, microglial activity, and synaptic function [3, 79, 83, 84]. However, brain, cerebrospinal fluid, and plasma lipid signatures are not equivalent, and most studies do not demonstrate that a microbiome‐induced eCBome change directly causes neuroinflammation.

In autoimmune and systemic inflammatory diseases, dysbiosis, barrier perturbation, and altered lipid mediators may influence T‐cell and myeloid programs [79, 83]. Current evidence is predominantly associative or mechanistically supportive rather than a complete causal chain. These conditions are therefore treated as systemic extensions rather than independent full disease modules.

Sex and age may be particularly relevant in these extensions because immune responses, microbial ecology, and lipid mediators differ across biological contexts [79, 83]. Studies should report sex‐disaggregated results and avoid extrapolating from male‐only animal models.

### 6.5 Cross‐Disease Evidence Synthesis

Across diseases, the recurring elements are altered microbial function, compartment‐specific lipid signaling, barrier or translocation changes, and immune‐cell activation [2, 52, 58, 59, 85, 86, 87]. Their recurrence does not prove a universal sequence. In some settings, barrier injury may precede eCBome remodeling; in others, inflammation, diet, or pharmacotherapy may alter both systems independently [52, 58, 59].

Evidence strength is therefore assigned using prespecified criteria rather than rhetorical confidence.

Strong evidence requires a defined microbial perturbation, direct eCBome measurement, a mechanistically linked barrier, immune or metabolic outcome, and replication or human support [19, 22, 33, 34, 36, 40, 52, 53, 58, 59]. Moderate evidence includes direct measurements but incomplete mediation, limited replication, or predominantly preclinical data. Emerging evidence is associative, compartmentally indirect, or based on extrapolation.

Using these criteria, gut permeability and metabolic endotoxemia provide the strongest mechanistic anchor; obesity‐associated inflammation is strong to moderate‐strong; IBD is moderate‐strong; MASLD/MASH is moderate; and neuroinflammatory or systemic autoimmune extensions remain emerging to moderate [19, 22, 33, 34, 36, 40, 59, 65, 66, 69, 74, 77, 78, 79, 83]. Table 3 presents the rationale and limitations rather than a purely subjective label.

**TABLE 3:** Disease evidence hierarchy using prespecified criteria.

Disease context	Evidence grade	Rationale and principal limitation
Gut permeability/metabolic endotoxemia	Strong	Defined microbiota perturbations, intestinal ECS measurements and barrier/LPS outcomes; human mediation remains less complete
Obesity/metabolic inflammation	Strong to moderate‐strong	Causal animal models plus human associations; central and peripheral CB1 effects and cohort heterogeneity complicate translation
Inflammatory bowel disease	Moderate‐strong	Human tissue and lipid data plus experimental colitis and engineered PEA delivery; limited disease‐modifying clinical evidence
MASLD/MASH	Moderate	Plausible gut‐liver and hepatic CB1 biology; few studies jointly measure microbial function, hepatic eCBome and longitudinal fibrosis
Neuroinflammation	Moderate/emerging	Strong independent microbiome and eCBome literatures; limited direct gut‐microbe‐to‐brain‐eCBome causal chains
Autoimmune/systemic inflammation	Emerging	Predominantly associative or convergent evidence with variable tissues, treatments, and disease mechanisms

*Note:* Evidence criteria: Strong = controlled microbial perturbation, direct eCBome measurement, linked downstream phenotype and replication or human support. Moderate = direct measurements with incomplete mediation, limited replication, or predominantly preclinical evidence. Emerging = associative, compartmentally indirect, or extrapolated evidence.

The rheostat is therefore best viewed as a testable cross‐disease architecture whose individual components must be demonstrated within each tissue and disease stage, not as a universal causal pathway.

## 7 Translational Strategies and Engineered Microbial Platforms

Microbiome‐based interventions differ substantially in composition, mechanism, regulation, and risk. Conventional probiotics are established food‐associated strains used without genetic engineering; next‐generation probiotics are newly characterized commensals selected for defined functions; genetically engineered probiotics are modified to sense or deliver specific molecules; defined live biotherapeutic products are regulated medicinal preparations with controlled identity and manufacturing; and postbiotics are nonliving microbial preparations or components. These categories should not be grouped as interchangeable “probiotics”.

Diet, prebiotics, and postbiotics can alter upstream substrates and microbial functions. Fiber supports SCFA production, whereas fat quality affects bile acids and fatty‐acid substrates for AEA, 2‐AG, and NAE synthesis [4, 5, 6, 24, 25, 28, 29, 30, 37, 88, 89]. Human dietary associations with circulating NAEs and microbial families are informative, but controlled interventions are needed to establish directionality (Table 4).

**TABLE 4:** Distinct therapeutic categories, mechanisms, and safety requirements.

Intervention category	Mechanism, evidence, and key considerations
Conventional probiotics	Established food‐associated strains; effects are strain‐ and host‐specific; avoid assuming class‐wide efficacy
Next‐generation probiotics	Newly characterized commensals selected for defined functions; require genomic, ecological, and safety characterization
Genetically engineered probiotics	Modified bacteria sense or deliver defined mediators such as PEA or OEA; require genetic stability, containment, and dose control
Defined live biotherapeutic products	Regulated medicinal live organisms with controlled identity, potency, and manufacturing; distinct from foods or supplements
Postbiotics	Nonliving microbial preparations or components; lower replication risk but composition and mechanism must be defined
Diet/prebiotics	Alter microbial substrates, SCFAs, bile acids and lipid precursors; adherence and background diet are major confounders
Peripheral CB1/CB2 strategies	Potential metabolic or immune modulation; central CB1 exposure carries psychiatric risk demonstrated by rimonabant
FAAH/MAGL modulation	Changes multiple lipid mediators; requires high selectivity, systems lipidomics and careful neurological safety assessment
PEA/OEA delivery	Related NAEs with distinct targets; oral bioavailability, tissue exposure, and mediator‐specific endpoints should be measured

Conventional and next‐generation probiotics may improve selected microbial or barrier functions, but effects are strain‐, dose‐, host‐, and disease‐specific [10, 11, 13, 84, 88, 90]. Safety assessment should include infection risk in vulnerable hosts, antimicrobial resistance, ecological persistence, and manufacturing consistency. Defined live biotherapeutics additionally require potency assays, genomic stability, containment, and regulatory‐grade production.

### 7.1 Engineered PEA‐ and OEA‐Producing Probiotics

Engineered *L. paracasei* F19 expressing human NAPE‐PLD provides one of the clearest causal platforms in the field. In toxin‐A‐induced colitis, palmitate‐supported PEA production improved histological injury, neutrophil and macrophage indices, cytokine and oxidative‐stress markers, and tight‐junction proteins; protection was abolished in PPARα‐deficient mice [72]. The combination of a defined bacterial construct, measured mediator production and receptor dependence directly links microbial engineering to eCBome signaling.

A related oleate‐regulated construct produced OEA in the intestine and improved metabolic, barrier, microbiota, and gut‐brain outcomes in high‐fat‐diet‐fed male mice [76]. These studies should be interpreted separately because PEA and OEA engage overlapping but distinct biology. Together with the next‐generation probiotic literature [91], they show how engineered bacteria can serve both as candidate therapeutics and as causal experimental tools [16, 23, 45, 46, 54, 72, 84].

### 7.2 Host‐Directed eCBome Pharmacology

Receptor and enzyme modulation remains attractive, but historical failures define the safety boundary. Rimonabant improved weight and cardiometabolic measures yet increased depressive and anxiety‐related adverse events; European authorization was suspended and withdrawn, establishing why centrally active CB1 antagonism is unsuitable for broad metabolic use [92]. Peripheral restriction, biased signaling and tissue‐selective delivery are therefore central design principles. CB2, PPARα, TRPV1, GPR55, and GPR119 remain potential targets, but receptor distribution and disease stage must guide interpretation [10, 16, 20, 21, 37, 84, 88, 93].

FAAH or MAGL inhibition can alter multiple lipids rather than one isolated mediator. The BIA 10‐2474 phase 1 catastrophe was associated with broad off‐target lipase inhibition and cortical‐neuron lipid‐network disruption, illustrating that compound selectivity and systems lipidomics are essential [10, 35, 84, 88, 94]. Clinical trials should therefore measure target engagement, compartment‐specific lipid changes, barrier and immune outcomes, and negative or null disease‐modifying endpoints rather than relying on symptom improvement alone.

## 8 Future Directions: A Causal and Compartment‐Resolved Roadmap

Future studies should use perturbations capable of falsifying the proposed pathway (Box 2). Gnotobiotic colonization and fecal‐community transfer can test community‐level effects; engineered bacteria and bacterial loss‐of‐function constructs can isolate a mediator‐producing function; receptor‐ or enzyme‐deficient hosts can establish dependence; and isotope tracing can connect microbial or dietary substrates to host lipid products [3, 19, 22, 88]. Multi‐omics integration is most informative when these perturbations are linked to prespecified mediation analyses rather than post hoc correlations [95, 96, 97, 98, 99].

BOX 2 **Priority causal experiments and unresolved questions.**

Which bacterial genes or metabolites alter specific eCBome ligands? Can isotope tracing connect microbial substrates to host NAEs or monoacylglycerols? Do bacterial loss‐of‐function constructs abolish the phenotype? Which epithelial, immune, or neural cell types are receptor dependent? How do sex, age, diet, circadian state, and medications modify the response? Can matched mucosal, plasma, and target‐tissue lipidomics predict disease activity or treatment response? Which engineered strains satisfy stability, containment, and regulatory requirements for human live biotherapeutics?

Tissue‐resolved lipidomics should quantify AEA, 2‐AG, PEA, OEA, and related lipids together with relevant enzymes and receptors [16, 98, 100, 101, 102]. Matched feces, intestinal mucosa, plasma or portal blood, and target tissues can distinguish local production from systemic redistribution. Sample collection should control fasting, diet, circadian timing, anesthesia, storage, and ex vivo lipid degradation.

Longitudinal human cohorts should determine whether microbial and eCBome changes precede disease activity, follow treatment, or persist during remission [96, 97, 98]. Repeated sampling should be integrated with diet, antibiotics, biologics, sex, age, and metabolic status.

Organoids, intestinal immune co‐cultures, and gut‐on‐chip systems can test epithelial and immune mechanisms with human cells, while animal models remain valuable for whole‐body physiology [2, 10, 72, 103, 104]. Trials should enroll biologically defined subgroups and combine microbial functions, expanded lipidomics, functional permeability assays, immune phenotyping, and disease‐specific outcomes [13, 14, 15, 88, 96, 97, 98]. Patient stratification should be validated prospectively rather than inferred from exploratory clusters [96, 97, 98]. Neuroimmune and autoimmune studies require particularly careful compartment matching and direct causal tests [13, 22, 83, 96, 97, 98, 101].

### 8.1 Minimum Evidence Set for a Causal Microbiome‐eCBome Claim

A strong causal claim should demonstrate: a controlled microbial perturbation; a measured change in a defined eCBome ligand, receptor, or enzyme; temporal precedence; blockade or loss of the downstream phenotype after bacterial, host‐receptor, or enzyme loss of function; and replication in a second model or human dataset [16, 21, 35, 100]. This minimum set converts the rheostat from a broad metaphor into a falsifiable experimental framework.

## 9 Conclusion

The microbiome‐eCBome axis is an established biological interface; the contribution of the rheostat framework is to make that interface measurable and evidence graded. Direct causality is strongest where microbial perturbation, eCBome signaling, barrier function, and metabolic inflammation have been measured within the same experiment, including engineered PEA‐ and OEA‐producing bacterial platforms. Translation will require compartment‐specific interpretation, explicit separation of classical endocannabinoids from related NAEs, validated barrier assays, balanced reporting of negative clinical evidence, and safety‐aware therapeutic design. A causal roadmap combining microbial genetics, receptor dependence, isotope tracing, human organoid, or chip systems and longitudinal multi‐omic trials can determine when the axis is a modifiable disease mechanism rather than an accompanying signature.

## Author Contributions

Reuben S. Maghembe: Conceptualization, methodology, project administration, supervision, visualization, writing – original draft, writing – review and editing. Donath Damian: Methodology, validation, writing – review and editing. Samweli Bahati: Methodology, visualization, writing – review and editing. Abdalah Makaranga: Validation, writing – review and editing. All authors reviewed and approved the final manuscript.

## Funding

The authors have nothing to report.

## Disclosure

Literature‐search and evidence‐appraisal note: This review was developed as a structured narrative synthesis and updated through 24 July 2026. PubMed/MEDLINE and Google Scholar were searched using combinations of microbiome, gut microbiota, endocannabinoid system, endocannabinoidome, AEA, 2‐AG, PEA, OEA, N‐acylethanolamine, FAAH, MAGL, inflammation, intestinal permeability, metabolic endotoxemia, IBD, obesity, MASLD/MASH, neuroinflammation, engineered probiotic, live biotherapeutic, and lipidomics. Backward citation tracing was used to identify primary mechanistic studies. Priority was given to human tissue or intervention evidence, controlled microbiota perturbations, direct eCBome measurements, receptor‐ or enzyme‐dependent models, compartment‐resolved lipidomics, and reproducibility. Evidence was classified as direct causal, mechanistically supportive, associative, or hypothesis‐generating and graded by human relevance, intervention strength, compartmental resolution, and replication. Because this was a narrative rather than systematic review, no PRISMA flow diagram or pooled effect estimate was generated.

## Conflicts of Interest

The authors declare no conflicts of interest.

## Data Availability

No new datasets were generated or analyzed in this review. All evidence discussed is derived from previously published literature.
